# The mediterranean dietary pattern and breast cancer risk in Greek-Cypriot women: a case-control study

**DOI:** 10.1186/1471-2407-12-113

**Published:** 2012-03-23

**Authors:** Christiana A Demetriou, Andreas Hadjisavvas, Maria A Loizidou, Giorgos Loucaides, Ioanna Neophytou, Sabina Sieri, Eleni Kakouri, Nicos Middleton, Paolo Vineis, Kyriacos Kyriacou

**Affiliations:** 1 Department of EM/Molecular Pathology, The Cyprus Institute of Neurology and Genetics, Nicosia, Cyprus; 2 Department of Epidemiology and Biostatistics, School of Public Health, Imperial College London, London, UK; 3 The Cyprus Institute of Neurology and Genetics, Nicosia, Cyprus; 4 Nutritional Epidemiology Unit, Istituto Nazionale Tumori, Milan, Italy; 5 Bank of Cyprus Oncology Center, Nicosia, Cyprus; 6 Department of Nursing, School of Health Sciences, Cyprus University of Technology, Limassol, Cyprus

## Abstract

**Background:**

Diet has long been suspected to impact on breast cancer risk. In this study we evaluated whether the degree of adherence to a Mediterranean diet pattern modifies breast cancer risk amongst Greek-Cypriot women.

**Methods:**

Subjects included 935 cases and 817 controls, all participating in the MASTOS case-control study in Cyprus. The study was approved by the Cyprus National Bioethics Committee. Information on dietary intakes was collected using an interviewer administered 32-item Food Frequency Questionnaire. Information on demographic, anthropometric, lifestyle, and other confounding factors was also collected. Adherence to the Mediterranean Diet pattern was assessed using two a-priory defined diet scores. In addition, dietary patterns specific to our population were derived using Principal Component Analysis (PCA). Logistic regression models were used to assess the association between the dietary patters and breast cancer risk.

**Results:**

There was no association with breast cancer risk for either score, however, higher consumptions of vegetables, fish and olive oil, were independently associated with decreased risk. In addition, the PCA derived component which included vegetables, fruit, fish and legumes was shown to significantly reduce risk of breast cancer (ORs across quartiles of increasing levels of consumption: 0.89 95%CI: 0.65-1.22, 0.64 95%CI: 0.47-0.88, 0.67 95%CI: 0.49-0.92, P trend < 0.0001), even after adjustment for relevant confounders.

**Conclusions:**

Our results suggest that adherence to a diet pattern rich in vegetables, fish, legumes and olive oil may favorably influence the risk of breast cancer. This study is the first investigation of dietary effects on breast cancer risk in Cyprus, a country whose population has traditionally adhered to the Mediterranean diet.

## Background

Breast Cancer (BC) is the most prevalent cancer among females worldwide, being responsible for more than 515 thousand deaths annually in all WHO regions [1]. Diet has long been suspected to affect BC risk and numerous studies have investigated its possible effect. Even though most of these studies conclude that any effect of diet on risk may be weak, the findings of some large scale prospective studies [2-10] emphasize the importance of further investigating the role of diet in BC etiology.

When investigating the effects of diet on BC, it is important to consider that individuals do not consume single foods, but combinations of several foods that contain both nutrient and non-nutrient substances. Given the complexity of human diets, the correlation and effect modification of intake of some nutrients, and the many nutrient-to-nutrient interactions, conclusions about the effect of consumption of a single nutrient, food group, or dietary constituent on a specific health outcome may be misleading. For these reasons, it is useful to examine patterns of nutrient intake that express several related aspects of dietary intake concurrently [11,12].

One such dietary pattern is the Mediterranean diet pattern. The Mediterranean diet has long been accepted as an example of a well-balanced diet but there is no gold standard "Mediterranean diet". At least 16 countries border the Mediterranean Sea and diets vary between these countries as well as also between regions within the same country. However, despite the above variation, a Mediterranean diet has the following common characteristics: high consumption of fruits, vegetables, bread and other cereals, potatoes, beans, nuts and seeds; low to moderate consumption of dairy products, fish, eggs and poultry; and infrequent consumption of red meat. In the Mediterranean diet, olive oil is frequently consumed and is an important mono-unsaturated fat source, and wine is consumed in low to moderate amounts.

The combination and range of foods included in this diet pattern, provide plenty of anti-oxidants such as flavonoids, carotenoids, and antioxidant vitamins, lots of phytochemicals including phytoestrogens, sufficient quantities of fiber, adequate folate, and a favourable fatty acid profile [10,13]. These nutrients have been linked to mechanisms of carcinogenesis and have been found [14], or are hypothesized to confer protective effects [15] on total cancer incidence, and more specifically on BC incidence. The hypothesis that adherence to a Mediterranean diet pattern decreases BC risk is also supported by the observation that countries bordering the Mediterranean Sea, which are more likely to adhere to such a diet, for example Greece, Spain, Italy and Cyprus, have the lowest BC incidence rates in Europe [1].

Adherence to the Mediterranean diet pattern in epidemiological studies is commonly assessed with the use of dietary scores, developed to capture how closely a subject's diet resembles the Mediterranean diet. In this study we evaluated whether the degree of adherence to a Mediterranean diet pattern is associated with a decreased BC risk amongst Greek-Cypriot women who participated in the MASTOS (Greek for "breast") study - to date, the largest case-control study carried out in Cyprus [16]. While it is commonly assumed that traditionally a large proportion of the island's population adheres to a Mediterranean diet pattern, the extent to which this is true is unclear, let alone whether, and which components, may be associated with a reduced BC risk, making first-time investigation of this issue among the Mediterranean island's female population invaluable.

## Methods

### Subjects

MASTOS was the first and to date largest BC case-control study to be carried out in Cyprus. Recruitment lasted for the three year period 2004-2006, and the main aim of the MASTOS study was to describe the frequency of established and recognized risk factors for BC among Cypriot women and to assess their association with BC occurrence. Information on the study design, data collection as well as initial analysis of MASTOS data with respect to BC risk factors is published elsewhere [16]. In summary, 1109 cases of breast cancer aged 40-70 years old and 1177 control women were recruited. Cases had a confirmed diagnosis of breast cancer between January 1999 and December 2005. Controls were women from the general population that attended the national mammography screening program and received a negative result. In the present report, the analysis was restricted to post-menopausal cases and controls only, since diet has been shown to have a different effect on pre- and post-menopausal BC [10,17,18].

### Data collection

Demographic and risk factor data were collected with the use of a specially designed questionnaire, through a standardized interview. The questionnaire included information on age, marital status, level of education, current and adolescent BMI, exercise status, smoking status, family history of breast or ovarian cancer, hormone use, and reproductive characteristics such as age at menarche, pregnancy, gestation period, parity, breastfeeding, age at first and last pregnancy. Weight, height and consequently BMI were based on actual measurements but all other information was self-reported. Exercise was taken to mean any form of regular recreational exercise, 2-3 times a week, for a minimum of 30 minutes each time.

In addition, a standardized diet interview was conducted with each subject using a Food Frequency Questionnaire (FFQ) designed to best capture consumption of food items whose contribution to the energy and nutrient intake of the Cypriot population is high. The resulting questionnaire was designed to capture an individual's habitual food intake over the previous year. Thirty two food items were included in the questionnaire under eight distinct categories i.e. fruit and vegetables, fish, meat, meat products and substitutes, cereals, dairies, agricultural products and sweets. Frequency of consumption for each food item was reported as number of portions daily, weekly or monthly, rarely consumed, or never consumed. Portion sizes for the foods consumed were assessed by showing subjects pictures of standard medium portions, decided on by experienced dieticians. The questionnaire also included questions on the kind of oil used while cooking, whether olive oil was used in salads, and what kinds of spreads were used on bread.

Adherence to the Mediterranean diet pattern was first assessed using the diet score proposed by Panagiotakos et al. [19]. The score was developed to incorporate the inherent characteristics of the Mediterranean dietary pattern and to examine its relationship with cardiovascular disease risk and biological markers [19]. Thus, the score includes the weekly consumption of the following 9 food groups: non-refined cereals, potatoes, fruit, vegetables, legumes, fish, red meat and products, poultry, full fat dairy products as well as use of olive oil in cooking, and alcoholic beverages. The scoring system for the level of consumption of each food group is described in Table 1 and the final score ranged from 0 to 55. Caloric intakes were not available due to the short nature of the FFQ and the grouping of several different foods in the same food group category. As a result, BMI and height were used as surrogate measures of the energy needs of each individual and were thus included and corrected for in all analyses. For the same reason, nutrient intakes could not be estimated with sufficient accuracy; therefore the diet scores chosen were based on food group intakes and did not involve any nutrient components.

**Table 1:** The Mediterranean Diet Score by Panagiotakos et al. as modified for the present analysis

How often do you consume:	Frequency of consumption (servings/week or otherwise stated)					
All cereals (bread, pasta, rice, etc.)	Never	1-6	7-12	13-18	19-31	> 32
	0	1	2	3	4	5
Potatoes	Never	1-4	5-8	9-12	13-18	> 18
	0	1	2	3	4	5
Fruits	Never	1-4	5-8	9-15	16-21	> 22
	0	1	2	3	4	5
Vegetables	Never	1-6	7-12	13-20	21-32	> 33
	0	1	2	3	4	5
Legumes	Never	< 1	1-2	3-4	5-6	> 6
	0	1	2	3	4	5
Fish	Never	< 1	1-2	3-4	5-6	> 6
	0	1	2	3	4	5
Red Meat Products	≤ 1	2-3	4-5	6-7	9-10	> 10
	5	4	3	2	1	0
Poultry	≤ 3	4-5	5-6	7-8	9-10	> 10
	5	4	3	2	1	0
Full fat dairy products (cheese, yogurt, milk)	≤ 10	11-15	16-20	21-28	29-30	> 30
	5	4	3	2	1	0
Use of Olive Oil (times/week)	Never	Rare	< 1	1-3	3-5	Daily
	0	1	2	3	4	5
Alcohol (ml/day)	0	< 300	400	500	600	> 700
	5	4	3	2	1	0

Some of the original diet score categories were modified for our analysis. First, the Cereals category was taken to include all cereals consumed, as information on types of cereal was not available. Unrefined and refined cereals differ mostly in their fiber content [20], therefore since nutrient measures were not estimated it was thought appropriate to group together all cereal consumption. In addition, use of olive oil in cooking was estimated to include use in cooking and as a salad dressing, as usage of olive oil in salads is the most common use of olive oil in Cyprus. Lastly, the Mediterranean Diet Score was first designed to assess the effect of diet on cardiovascular disease. Since some alcohol consumption is beneficial to cardiovascular health, in the original score, zero alcohol consumption received a low score. However, for cancer, any amount of alcohol is detrimental to cancer risk [8]. Therefore, for our purposes zero alcohol consumption, received the highest score.

Secondly, the diet score developed by Martinez-Gonzalez et al. [21] was used. The score was developed as a quicker and simpler tool to estimate quantitatively adherence to the cardioprotective elements of the Mediterranean diet [21]. Nine items are included in the diet score and scored as shown in Table 2. The resulting score ranged from 0 to 9 points.

**Table 2:** Dietary Items included in the Martinez-Gonzalez Score as modified for the present analysis

	Yes
**1**. Olive Oil (≥ 1 spoon/day)	+1
**2**. Fruit (≥ 1 serving/day)	+1
**3**. Vegetables *or *Salad (≥ 1 serving/day)	+1
**4**. Fruit (≥ 1 serving/day) *and *Vegetables (≥ 1 serving/day)^**a**^	+1
**5**. Legumes (≥ 2 servings/week)	+1
**6**. Fish (≥ 3 servings/week)	+1
**7**. Any alcohol (zero consumption)	+1
**8**. Meat (< 1 serving/day)	+1
**9**. All cereals (bread, rice etc.) (1-3 servings/day)	+1

^**a **^One point is added when ≥ serving/day of both fruits and vegetables is consumed

Again, some of the original score components were modified. Wine consumption (component 7) was substituted by any alcohol consumed. Furthermore, since this score was also originally designed to assess diet's effects on cardiovascular health, the scoring was reversed to score +1 if no alcohol was consumed. Lastly, one of the characteristics of the Mediterranean diet is high consumption of cereals including pasta and potatoes. Therefore, component nine included pasta, rice and potatoes. For the purposes of this analysis consumption of one to three portions of any cereal daily was scored with +1 and all other consumption (i.e. lower or higher) was scored with zero.

### Statistical analysis

Differences between socio-demographic characteristics, potential risk factors (including overall diet scores) between cases and controls were initially explored using chi-squared or *t*-test as appropriate (e.g. height). In order to assess the association between levels of adherence to a Mediterranean diet and BC, adjusted Odds Ratios (ORs) of BC risk were estimated (a) across categorical levels and (b) per 1 unit increase in either of the diet scores in multivariable logistic models after adjusting for the possible confounding effect of the risk factors that best predict BC risk in this population [16]. In addition to the overall scores, ORs of BC per 1 unit increase in servings/week (or servings/day accordingly) were also estimated for each single components of the diet scores.

To further examine the dietary intakes of the Cypriot population, factor analysis with principal component extraction (PCA) was performed in order to derive the patterns that best describe the dietary habits of our population. It was deemed appropriate to restrict this analysis to the control group. Dietary intakes (in g/month for food items and ml/month for drink items) of each of the 32 food and beverage items in our FFQ were considered. Olive oil was not entered as a variable in PCA because only frequency and not quantity of consumption was assessed in the FFQ. Extraction of principal components was followed by a diagonal (oblimin) rotation, following failure of orthogonal rotations to produce interpretable results. For each dietary pattern identified, a component score was calculated for each subject using all factor loadings and the respective consumption of each food and beverage item. The association between BC and each of the observed factors were investigated in multivariable logistic models after mutually adjusting for all others. All statistical analyses were performed using STATA statistical software, version 11 (StatCorp. 2007. College Station, TX) and SPSS statistical software version 17.0 (PASW Inc.)

## Results

The characteristics of the 1752 post-menopausal women (among a total of 2286, 76.6%) participating in the MASTOS case-control study are presented in Table 3. As expected, compared to controls, cases tend to be more educated, of a larger BMI, an older age at FFTP, more likely to have a family history of BC, less likely to have breastfed their children, more likely to have smoked and less likely to have exercised more than 6 hours per week prior to diagnosis. However, contrary to what was expected, cases were younger and less likely to have used HRT. Cases and controls did not differ in terms of height, age at menarche, parity. Furthermore, no association was observed between case/control status and either of the two diet scores investigated here.

**Table 3:** Sociodemographic characteristics and potential risk factors among post-menopausal participants of the MASTOS case control study

Variable	Controls (% of n = 817)^a^	Cases (% of n = 935)^a^	P-Value
Education Category			
- Primary	395 (48.3)	394 (42.1)	0.044
- Secondary	277 (33.9)	334 (35.7)	
- College	77 (9.4)	98 (10.5)	
- University	62 (7.6)	96 (10.3)	

Age at Interview			
- < 45 years	4 (0.5)	57 (6.1)	< 0.001
- 45-54 years	187 (22.9)	263 (28.1)	
- 55-64 years	488 (59.7)	383 (41.0)	
- ≥ 65	138 (16.9)	232 (24.8)	

Height	158.7 ± 6.2	159.1 ± 7.1	0.190^b^

BMI category			
- < 20 (kg/m^2^)	6 (0.7)	20 (21.4)	0.001
- 20-24.9 (kg/m^2^)	190 (23.3)	216 (23.1)	
- 25-29.9 (kg/m^2^)	323 (39.5)	326 (34.9)	
- ≥ 30 (kg/m^2^)	201 (24.6)	303 (32.4)	

Family History of BC			
- No	750 (91.8)	770 (82.4)	< 0.001
- Yes	66 (8.1)	163 (17.4)	

Age at Menarche			
- ≥ 14	326 (39.9)	331 (35.4)	0.087
- 12-13	400 (49.0)	477 (51.0)	
- > 12	87 (10.6)	123 (13.2)	

Parity			
- 0	65 (8.0)	88 (9.4)	0.251
- 1	56 (6.9)	68 (7.3)	
- 2	330 (40.4)	403 (43.1)	
- 3 or more	366 (44.8)	376 (40.2)	

Age at FFTP^c^			
- < 20	108 (13.2)	92 (9.8)	0.007
- 20 - < 25	378 (46.3)	397 (42.5)	
- 25 - < 30	189 (23.1)	236 (25.2)	
- ≥ 30	123 (15.1)	185 (19.8)	

Breastfeeding			
- Yes	574 (70.3)	606 (64.8)	0.015
- No	243 (29.7)	329 (35.2)	

Oral Contraceptive use			
- No	679 (83.1)	758 (81.1)	0.352
- Yes	137 (16.8)	172 (18.4)	

HRT use			
- No	572 (70.0)	794 (84.9)	< 0.001
- Yes	238 (29.1)	133 (14.2)	

Smoking Status			
- Never	676 (82.7)	747 (79.9)	0.003
- Past	53 (6.5)	102 (10.9)	
- Current	88 (10.8)	84 (9.0)	

Exercise			
- No exercise	399 (48.8)	523 (55.9)	< 0.001
- Up to 3 hrs/wk	214 (26.2)	252 (27.0)	
- > 3 and ≤ 6 hrs/wk	141 (17.3)	127 (13.6)	
- > 6 hrs/wk	63 (7.7)	33 (3.5)	

Mediterranean Diet Score - Panagiotakos			
- ≤ 30	227 (27.8)	292 (31.2)	0.264
- 31-35	224 (27.4)	266 (28.4)	
- 36-40	239 (29.3)	246 (26.3)	
- > 40	127 (15.5)	131 (14.0)	

Mediterranean Diet Score - Martinez-Gonzalez			
- 0-4	258 (31.6)	322 (34.4)	0.429
- 5-6	237 (29.0)	242 (25.9)	
- 6-7	196 (24.0)	221 (23.6)	
- 8-9	126 (15.4)	150 (16.1)	

^a ^Failure of category counts to add up to this number, or of percentages to add up to 100 denotes missing values

^b ^Mean (SD) and p-value of independent t-test. In all other cases, p-values of χ^2 ^test are presented.

^c ^First full-term pregnancy

However, the distribution of cases and controls across the diet score categories indicated that the Mediterranean diet pattern is adhered to by a large percentage of Cypriots. More than 40% of the Cypriot population has a score of above 35 using the Panagiotakos Mediterranean diet score, and close to 40% of the Cypriot population scores above 6 using the Martinez-Gonzalez score.

### Mediterranean diet score by Panagiotakos

The mean intake of the 11 food indices used to construct the Mediterranean Diet score by Panagiotakos among post-menopausal cases and controls is shown in Table 4. Risk of BC decreased in association with high intake of vegetables and salads (OR per unit increase in servings/week: 0.95, 95% CI: 0.92-0.99), fish (OR per unit increase in servings/week: 0.88, 95% CI: 0.79-0.98), and olive oil (OR per unit increase in servings/week: 0.95, 95% CI: 0.92-0.99). A unit increase in the overall diet score by Panagiotakos was not associated with risk of post-menopausal BC (Table 4). Similarly, when treated as a categorical variable, divided in quartiles, this diet score was not associated with BC risk (OR Q4 vs. Q1: 0.99 95% CI: 0.70-1.40) (Table 4).

**Table 4:** Mediterranean Diet Score by Panagiotakos et al. and Post-Menopausal BC risk

	Intake^a, b^		
Food Indexes	Post-menopausal cases (n = 934)	Post-menopausal controls (n = 815)	Adjusted OR per unit increase (95% CI)^c^
All cereals	18.7 ± 7.8	19.4 ± 8.8	0.99 (0.98, 1.00)
Potatoes	2.0 ± 1.4	1.9 ± 1.3	0.95 (0.87, 1.03)
Fruits	9.2 ± 6.0	9.6 ± 6.9	1.00 (0.99, 1.02)
Vegetables	5.8 ± 2.8	6.2 ± 3.4	0.95 (0.92, 0.99)*
Legumes	2.1 ± 1.2	2.2 ± 1.4	0.95 (0.88, 1.03)
Fish	1.6 ± 1.1	1.8 ± 1.2	0.88 (0.79, 0.98)*
Red meat and products	2.8 ± 2.1	2.7 ± 2.1	0.98 (0.93, 1.03)
Poultry	1.6 ± 0.9	1.7 ± 0.9	0.91 (0.81, 1.04)
Full fat dairy products	6.5 ± 6.2	7.3 ± 6.8	0.98 (0.96, 1.00)
Use of olive oil (times/week)	6.6 ± 2.9	7.0 ± 3.4	0.95 (0.92, 0.99)*
Alcohol	16.7 ± 43.1	18.1 ± 49.5	1.00 (1.00, 1.00)
Mediterranean Diet Score	35.5 ± 3.1	35.8 ± 2.9	1.00 (0.96, 1.04)
Mediterranean Diet Score Across quartile levels			
- Q1: ≤ 34			1.00
- Q2: 35-36			1.02 (0.76, 1.36)
- Q3: 37-38			1.00 (0.75, 1.34)
- Q4: > 38			0.99 (0.70, 1.40)
			p-value for trend = 0.06

Levels (means ± SDs) of the Mediterranean Diet Score food indices in post-menopausal breast cancer cases and post-menopausal controls and adjusted odds ratios (with 95% CI) of BC in terms of the overall score and each its components

^a ^All values are means ± SDs

^b ^Servings/week or as otherwise stated

^c ^Adjusted for the following covariates: age at interview, family history, age at FFTP, HRT use, exercise, age at menarche, height and BMI in post-menopausal women

### Mediterranean diet score by Martinez-Gonzalez

The mean intake of the 9 food indexes used to construct the Mediterranean Diet score by Martinez Gonzalez among post-menopausal cases and controls is shown in Table 5. Similar to what was observed with the diet score by Panagiotakos, risk of BC decreased in association with high intake of vegetables and salads (OR per unit increase in servings/day: 0.74, 95% CI: 0.57-0.96), fish (OR per unit increase in servings/week: 0.88, 95% CI: 0.79-0.98), and olive oil (OR per unit increase in spoons/day: 0.76, 95% CI: 0.59-0.97). However, a unit increase in the diet score by Martinez-Gonzalez did not significantly increase or decrease risk of post-menopausal BC (Table 5). Similarly, when treated as a categorical variable, in quartiles, this diet score was not associated with BC risk (OR Q4 vs. Q1: 0.99 (0.70-1.40).

**Table 5:** The Martinez-Gonzalez Diet Score and Post-Menopausal BC risk

	Intake^a^		
**Food Indexes**	**Post-menopausal cases (n = 934)**	**Post-menopausal controls (n = 815)**	**Adjusted OR per unit increase (95% CI)^b^**

Olive oil (servings/day)	1.1 ± 0.4	1.1 ± 0.5	0.76 (0.59, 0.97)*

Fruit (servings/day)	1.3 ± 0.8	1.3 ± 0.9	1.01 (0.89, 1.15)

Vegetables or Salad (servings/day)	0.8 ± 0.4	0.9 ± 0.5	0.74 (0.57, 0.96)*

Fruit AND Vegetables (servings/day)	2.1 ± 1.0	2.2 ± 1.1	0.96 (0.86, 1.06)

Legumes (servings/week)	2.1 ± 1.2	2.2 ± 1.4	0.94 (0.87, 1.02)

Fish (servings/week)	1.6 ± 1.1	1.8 ± 1.2	0.88 (0.79, 0.98)*

Meat (servings/day)	0.6 ± 0.3	0.6 ± 0.3	0.73 (0.54, 1.04)

Cereals (servings/day)	2.9 ± 1.1	3.0 ± 1.2	0.93 (0.85, 1.02)

Alcohol (glasses/day)	0.1 ± 0.3	1.1 ± 0.4	0.99 (0.74, 1.31)

Martinez-Gonzalez Diet Score	5.5 ± 1.9	5.6 ± 1.9	1.00 (0.95, 1.06)

Martinez Gonzalez Diet Score			
Across quartile levels			
- Q1: 0-4			1.00
- Q2: 5-6			0.25 (0.64, 1.13)
- Q3: 6-7			0.84(0.72, 1.30)
- Q4: 8-9			0.63 (0.77, 1.53)

			p-value for trend = 0.61

Levels of the Martinez-Gonzalez Diet Score food indices in post-menopausal breast cancer cases and post-menopausal controls and adjusted odds ratios of BC (with 95% CI) in terms of the overall score and each of its components

^a^All values are means ± SDs

^b ^Adjusted for the following covariates: age at interview, family history, age at FFTP, HRT use, exercise, age at menarche, height and BMI in post-menopausal women only.

### Principal component analysis

Based on an eigenvalue criterion of > 1.0, and scree plot analysis 11 components were initially observed. However, factor interpretability justified the retention of only 4 components, as components 5 to 11 showed high factor loadings on single variables. As a result we repeated the technique, retaining and rotating only four factors. Each of the retained components was labeled according to the dietary pattern they represented: Meat/Potatoes (pattern 1), Cereals/Milk/Dairy (pattern 2), Cakes/Sweets/Nuts/Crackers/Pasta/Rice (pattern 3) and Fruit/Vegetables/Fish/Legumes (pattern 4) (Table 6). The four factors accounted for 23.6% of the total variance in the 32 food and beverage items (8.05, 5.92, 5.10, 4.55% for the 4 patterns respectively).

**Table 6:** Principal Component Analysis Dietary Patterns

Food	Pattern 1: Meat/Potatoes	Pattern 2: Cereals/Milk/Dairy	Pattern 3: Cakes/Sweets/Nuts/Crackers/Pasta/Rice	Pattern 4: Fruit/Vegetables/Fish/Legumes
Red Meat	0.568			

Poultry	0.447			

Deli	0.445			

Potatoes	0.414			

Sausages	0.355			

Rabbit	0.342			

Yogurt Light		0.593		

Cheese Diet		0.546		

Milk Skimmed		0.430		

Cheese Low-fat		0.388		

Milk Full-fat		0.345		

Cheese Full-fat		0.342		

Cereals		0.342		

Cakes			0.621	

Chocolates			0.542	

Bakery			0.450	

Nuts			0.368	

Crackers/Biscuits			0.362	

Pasta/Rice			0.345	

Fish fillet				0.565

Vegetables/Salad				0.528

Fruit				0.447

Legumes				0.415

Fish other				0.336

Factor loadings > 0.340 based on Principal Components Analysis of average intake of 32 food and beverage items, standardized and followed by diagonal rotation

Pattern 4 more closely resembles the Mediterranean diet pattern. This was further analyzed to determine its association to BC risk. Quartile values for Pattern 4 based on the distribution of controls, were used to divide cases and controls with the highest score quartile assigned a score of 4, while the lowest score quartile were assigned a score of 1. Quartiles of adherence to this dietary pattern were entered into a multivariate regression model adjusting for all potential confounders, to determine whether high adherence to the Fruit/Vegetables/Fish/Legumes dietary pattern decreases BC risk. Since, in the oblique rotation, the factors are allowed to be correlated, in the multivariable regression model we also adjusted for the other three PCA derived patterns, also as categorical variables. It was found that post-menopausal BC risk decreased across quartile levels of increased adherence to the fourth dietary pattern (OR Q4 vs. Q1: 0.67 95% CI: 0.49-0.92, P for trend < 0.0001) (Table 7). Patterns 1, 2, and 3 were not associated with BC risk whether treated as continuous or categorical variables, and there was no evident trend (data not shown).

**Table 7:** Odds Ratios of BC in relation to Principal Component Analysis-derived pattern 4

	Cases/Controls	Adjusted OR^**a**^	95% Confidence Interval
**Pattern 4: Fruit/Vegetables/Fish**			

Quartile 1	261/174	1.00	
Quartile 2	251/192	0.89	0.65-1.22
Quartile 3	213/222	0.64	0.47-0.88
Quartile 4	208/226	0.67	0.49-0.92
P for trend		< 0.0001	

Quartiles of adherence to dietary pattern 4, derived from Principle Component Analysis, in relation to BC risk

^**a **^Odds Ratio adjusted for the following covariates: age at interview, family history, age at FFTP, HRT use, exercise, age at menarche, height, BMI and PCA derived patterns 1,2 and 3 in post-menopausal women only

## Discussion

This study was the first to investigate dietary patterns in relation to BC risk in Cyprus, an island where the diet closely resembles the Mediterranean diet, as shown by the relatively large percentage of Cypriots that receive high scores of adherence to the Mediterranean diet patterns examined. None of the two Mediterranean Diet scores employed in our analysis were found to be associated with BC risk. However, in both scores, specific food indexes - Vegetables, Fish and Olive Oil - were inversely associated with BC risk. Although the individual effect estimates observed are small, if one assumes that the positive effects of different food groups are additive, then the reduction in BC risk through a diet that includes all the beneficial components, could be substantial. This combined effect becomes obvious when the fourth PCA dietary pattern, a combination of the protective components, is analyzed. Further, considering the high incidence of BC and assuming a causative association, even a small reduction in risk conferred by dietary components can be translated into several prevented cases per year, in Cyprus alone.

The application of Mediterranean diet scores in epidemiological studies has yielded conflicting results. Recently a study in the EPIC cohort, demonstrated that higher adherence to a Mediterranean dietary pattern - as assessed using the Mediterranean diet score developed by Trichopoulou [22] - is associated with a decreased cancer risk both in Mediterranean and in non-Mediterranean countries (HR: 0.96, 95% CI 0.96-0.98 for a two point increment in the score). This analysis did not identify any of the components of the score as having a predominant effect, supporting the idea that it is the combination and range of nutrient and non nutrient components of the diet, that confer the protective effect [13]. In a more focused analysis of the EPIC cohort with respect to BC risk, adherence to the Mediterranean diet pattern - assessed using an adaptation of the Trichopoulou score - was found to confer a small decrease in total and post-menopausal BC risk. This protective effect became stronger when no consumption of alcohol - instead of moderate -was treated as most beneficial and was more pronounced for ER-/PR- post-menopausal tumours [10]. A study on the Greek EPIC cohort has also demonstrated a marginally significant inverse association between adherence to a Mediterranean Diet pattern (defined a priori) and post-menopausal BC [23]. However, Cade et al. [24] who examined the adherence to a Mediterranean diet pattern in a British cohort did not yield any significant results, except a non significant trend towards decreased risk, with increasing adherence in pre-menopausal women. This cohort included women with different dietary patterns and at extremes of intake, so as to enable comparisons and maximize the potential of successful identification of any interactions. Similarly, Fung et al. [2] investigated post-menopausal BC risk in the Nurses' Health Study, but found no significant association with adherence to the Mediterranean diet, however results for ER- BC approached significance. A meta-analysis of 6 prospective studies with a total of 512,366 subjects followed for a time ranging from eight to 18 years, showed that adherence to the Mediterranean diet is inversely associated with total cancer incidence and mortality, but there were no results specific to BC [25].

These conflicting findings might be explained by the limitations of diet scores such as the subjectivity associated with which food items are included in each score and how each component is scored. The score may be missing food items that largely determine the dietary habits of the population under study. Also, if the cut offs used for scoring are based on median values from a particular population, it is unlikely that they will yield useful and interpretable results, in a different population with different dietary habits. Another explanation for the contradictory findings is that traditional methods of food preparation vary widely within and between countries. It is argued that results from Northern European countries may differ from results from Mediterranean populations because essential components of the Mediterranean diet may be consumed in different forms and proportions in these two regions [24].

In order to derive dietary patterns that best reflect the dietary habits of the Cypriot population, PCA was applied to the dietary data, yielding four dietary patterns. The fourth dietary pattern - Fruit/Vegetables/Fish/Legumes - included both vegetables and fish that were found to be inversely associated with BC risk in both diet scores. In the MASTOS study, vegetable and fish intakes are correlated with olive oil consumption (0.9705 and 0.2180 respectively, both significant at the 0.05 level), therefore Pattern 4 can also be taken to include olive oil use despite the food's absence from the 32 FFQ variables entered into PCA. High adherence to this dietary pattern was found to decrease BC risk in post-menopausal women and can be recommended to women as a healthy dietary pattern which may indeed reduce BC risk.

It should be noted that PCA, is not without limitations since there is subjectivity associated with the derivation of the variables to be included, the number of factors extracted, the rotation used, and the labelling of the factors retained. Despite these, most studies applying PCA on their dietary data and deriving a dietary pattern similar to ours, demonstrated significant reductions in BC risk. While Velie et al. [12] found that a vegetable-fish/poultry-fruit diet pattern was not significantly correlated with BC, Ronco et al. [26] found that adherence to a food pattern rich in anti-oxidants - associated with white meat, fruit, vegetables - was protective in a study carried out in Uruguay. Murtaugh et al. [27] in their study of Hispanic and non-Hispanic white women, evidenced that a dietary pattern labeled as Mediterranean was statistically significant showing an inverse association with BC. In the French subgroup of the EPIC cohort, a "healthy/Mediterranean" (essentially vegetables, fruits, seafood, olive oil, and sunflower oil) diet pattern, was significantly inversely associated with BC risk, especially for ER+/PR- cancers [28]. Lastly, in the ORDET cohort, a dietary pattern which was characterized by high consumption of raw vegetables and olive oil as added fat, was found to significantly decrease BC risk [29].

The variables loading on the fourth PCA-derived pattern presented here have yielded varying results in studies investigating their relationship to BC. Concerning vegetable intake, most studies on post-menopausal women gave no significant findings [30-35]. However, Sonestedt et al. in an analysis of the Malmo Diet and Cancer Cohort demonstrated that in post-menopausal women with a BMI less than 27 kg/m^2 ^and in post-menopausal women not changing their diet before recruitments, there was a significant reduction in BC risk with increased intake of fruit, berries and vegetables [36]. Also, analysis of the Greek cohort of the EPIC study evidenced that a high consumption of vegetables and fruits was one of the most beneficial components, for the protective effect of the Mediterranean diet on survival [3]. In addition, in the EPIC study after a mean of 11 years of follow up, there was a significant reduction in BC incidence, associated with higher intake of vegetables [10]. Studies on fish intake again yielded mostly no significant results [37-43]. The only statistically significant result concerning fish intake comes from the Singapore Chinese Health Study, which evidenced that intake of marine n-3 fatty acids, had a protective effect on BC incidence [44]. Lastly, concerning olive oil intake, a meta-analysis of 13800 patients and 23340 controls in 19 observational studies, evidenced that olive oil intake is inversely associated with BC risk [18]. In light of these findings, vegetables, fish, and olive oil may work synergistically in a dietary pattern to decrease BC risk, as was shown by our results.

Having discussed the significance of our findings in the context of the available literature, the limitations of this study should be mentioned. Cases included in the analysis were diagnosed over a seven year time frame, immediately before the conduction of the study. This may have introduced a survival bias into our study, but the 95% 10-year survival rate observed at the cancer referral centres in Cyprus [45] suggests that this bias is likely small. In addition, response rate was 98%, therefore selection bias other than survival is not a big concern. Further, our FFQ was limited since it examined intake of only 32 food items and there is no information about the way each food is consumed. Perhaps of most concern is the problem of measurement error in dietary assessment, particularly associated with the use of FFQs in case-control studies. However, it is unlikely that cases differentially recalled the specific food groups that were included in the diet scores or the dietary patterns derived from PCA. In addition, because diet is not an established BC risk factor, differential misclassification is less likely. On the other hand, both cases and controls are likely to have underreported their intakes, a common concern of FFQ use. However, such bias is non differential which tends to bias any findings towards the null, thus suggesting that the true effects might be stronger than those observed. Lastly, the four components retained from PCA only explained 23.6% of the variance, but this is comparable to other dietary studies, reflecting the challenges associated with reducing highly interrelated and complex dietary variables [46].

Despite the limitations associated with a case-control study design, these are the only data available in Cyprus since a cohort study has not been yet established in our population. Considering the location of the country and the dietary habits of the population that closely resemble the traditional Mediterranean diet, an investigation of this diet's effects on BC was warranted. Also, the sample size of this study ensures adequate power to delineate diet's effects on BC risk.

In addition to the association with BC risk, in our analysis there was a significant inverse association between adherence to the fourth PCA dietary pattern and age at interview (P value for chi-square test: 0.004). Thus, in the future, it will be interesting to investigate how the shift towards or away from this healthy dietary pattern in the younger generations impacts on BC incidence, as well as on other BC associated parameters.

## Conclusion

In conclusion, our results suggest that adherence to a diet pattern rich in vegetables, fish, legumes and olive oil may favorably influence the risk of BC by having a protective effect. Despite the weak individual effect estimates, when these healthy food groups are combined into a dietary pattern the effect estimates obtained are larger and suggest that they act synergistically to confer a substantial reduction in BC risk. Given this protective effect in post-menopausal women, such a dietary pattern can be recommended to women as a healthy dietary pattern which may indeed contribute to a reduction in BC risk. This study is the first investigation of dietary effects on BC risk in Cyprus, a country whose population has traditionally adhered to the Mediterranean diet. Additional cohort studies of dietary patterns in our as well as in other Mediterranean populations are needed, to further clarify the protective effects of this diet on BC risk.

## Competing interests

The authors declare that they have no competing interests.

## Authors' contributions

CAD performed the statistical analysis and interpretation of the dietary data and drafted the paper. AH MAL GL and KK have made substantial contributions to conception and design of the study and in the acquisition of data. They have also been involved in drafting the manuscript and revising it critically for important intellectual content. EK made substantial contributions to the acquisition of data. IN SS NM and PV were involved in drafting the manuscript and revising it critically for important intellectual content. All authors have given their approval for the submitted version.

## Pre-publication history

The pre-publication history for this paper can be accessed here:

http://www.biomedcentral.com/1471-2407/12/113/prepub
